# Performance Evaluation of Asphalt-Pavement Crack-Repair Materials

**DOI:** 10.3390/ma18071611

**Published:** 2025-04-02

**Authors:** Congying Liu, Hongchang Wang, Song Liu, Dagang Yang, Yue Xiong

**Affiliations:** 1 School of Civil Engineering, Nanjing Forestry University, Nanjing 210037, China; 13852154483@163.com (C.L.); ls2020@njfu.edu.cn (S.L.); m15996206878@163.com (D.Y.); 13547678738@163.com (Y.X.); 2 Jiangsu Highway Intelligent Detection and Low-Carbon Maintenance Engineering Research Center, Nanjing 210037, China

**Keywords:** crack repair, asphalt pavement, performance evaluation, indoor test, finite-element analysis

## Abstract

Based on the requirements for asphalt pavement crack repair materials, five representative materials were selected for testing: type-A crack sealant, type-B crack sealant, 70# hot asphalt, SBS-modified asphalt, and ambient-temperature water-based crack filler. A series of material performance and pavement performance experiments were conducted on these materials. Additionally, numerical models were developed based on actual asphalt pavement crack repair structural conditions. Under the ambient temperatures of 0 °C, 20 °C, and 50 °C, considering two types of loads, namely static load and dynamic load, the shear stress, tensile stress, and compressive stress of the crack-repair structure were analyzed in detail. The stress state of the repaired structure was specifically examined under the most unfavorable load conditions. These analyses were validated by comparing with laboratory-measured stress data, providing important references for the application of asphalt pavement repair materials. The conclusion of the research indicates that the B-type grouting adhesive, as a special material for crack repair, has obvious advantages in shear and tensile strength, and its overall performance is the best. It is suitable for expressways, first-class roads, and urban expressways. Asphalt materials for heating construction have obvious economic advantages compared with special materials and are suitable for low-grade asphalt pavement with relatively small pressure and small traffic volume on highways, branch roads, and secondary roads. Normal-temperature construction is suitable for temporary repair under adverse conditions such as cracks and dampness after rain.

## 1. Introduction

In recent years, “reconstruction light maintenance” of the wrong concepts brought about by the problem of road workers has attracted the attention of road workers [1], as asphalt pavement crack repair; maintenance; and many other aspects of the problem, such as maintenance specifications, repair processes and materials, etc., are in urgent need of systematic and in-depth research [2,3,4]. Kun Xiong adds mineral powders to polyurethane grouting materials to provide a more economical and durable solution for repairing cracks in asphalt pavements [5]. Franesqui et al. demonstrated the suitability of metallic fillers over mineral powders as additives for microwave self-healing pavements and the feasibility of ultrasonic tracking of crack depths [6]. Zhu et al. studied self-adhesive basalt fiber geotextile (SBFG) consisting of basalt fiber cloth hot-melt laminated with modified asphalt for inhibiting and controlling reflective cracks in asphalt pavements [7]. Tabakovic et al. proposed a new method of incorporating a segregated calcium alginate fiber encapsulating an asphalt binder into asphalt pavement mixtures [8]. Franesqui et al. proposed a calibrated model for ultrasonic inspection of different types of asphalt mixtures (semi-compact asphalt concrete AC-S, stone-mastic asphalt SMA, and porous asphalt PA) for assessing the depth and extent of macroscopic cracks in asphalt pavements that extend from top to bottom [9]. Li Feng, Huang Songchang, and others designed the industry standard “Pavement Heating Sealant” (JT/T 740-2015) [10]. Sun Huadong et al. established of a cohesion modeling system and method for evaluating the adhesion performance of grouting materials [11]. In order to alleviate the deformation accumulation caused by cyclic loading and external temperature when ordinary asphalt is used as crack repair material, Song developed shape memory epoxy resin (SMEP)/(SBS) composite-modified asphalt (SMEA) with good shape memory and road performance [12]. Wang investigated the effect of cyclic temperature changes on the crack-repair structure of asphalt pavement, and the results showed that the bond interface would be subjected to tensile and shear stresses under the action of cyclic temperature changes [13]. From the discussion of road problems at home and abroad, the development of crack repair technology has achieved more results. There are three main development directions, one of which is the development of crack materials to make them have a better performance [14,15]; the second is the development of crack construction technology to effectively strengthen the quality of the project [16,17,18]; and the third is to improve the detection and analysis ability of asphalt pavement cracks to prevent disease extension and prolong pavement life [19,20,21,22,23]. The current research on crack repair technology is relatively limited, and despite the rapid development of high-performance grouting materials (especially low-temperature performance materials), their selection lacks clear technical indicators and relies only on workers’ experience, which seriously restricts the standardization and reliability of the repair work. Thus, it is necessary to research grouting materials, performance characteristics, and the scope of application of the investigation to better ensure the quality of grouting in order to prolong the asphalt pavement structure and reduce the maintenance time. Therefore, to fill the gap in repair material selection criteria, it is necessary to explore the performance characteristics and applicable range of grouting materials to better ensure the quality of grouting and extend the service life of asphalt pavement repair structures.

This paper considers the above factors to select five representative repair materials, through an indoor test on its comprehensive performance and scope of application assessment, and finally according to the asphalt-pavement crack-repair structure of the most unfavorable conditions for modeling and stress calculation, as well as an indoor test-measured stress-value comparison to verify the repair materials in extreme conditions of the road performance for the actual application of asphalt pavement repair materials to provide a reference basis.

## 2. Material Property Requirements

The performance characteristics of joint-filling materials should meet three requirements:

(1) It is required that the joint-filling materials meet many requirements in terms of performance, such as high-temperature stability, corrosion resistance, crack resistance, aging resistance, etc., to ensure the quality of joint filling, that it is adapted to the local temperature environment [24], and that it can maintain stability under conditions such as oil leakage corrosion and have strong resistance to foreign body embedding;
(2) It is required that the filling material meet the requirements of local working conditions and has good construction performance;
(3) The road performance of the repaired joint structure should meet the specifications and other standards to ensure that the road performance and strength performance meet the requirements, increase the service life of the road surface, and improve the driving safety and experience.

To this end, it is necessary to conduct high-temperature stability (softening point and fluidity test), low-temperature crack resistance (5 °C ductility and elastic recovery test), anti-foreign body embedding (cone penetration test), corrosion resistance (corrosion resistance test), and anti-aging test. This item is special because it cannot provide direct detection items to obtain evaluation data. The scheme adopted in this paper is to provide a 60 °C oven and place the material to be tested in it for 5 days. Its function is to simulate the thermal oxygen aging process under experimental conditions and compare the obtained results with experimental results of the original material to measure its anti-aging level. The comprehensive properties of all kinds of joint-filling materials were evaluated correctly by experimental research on the shear and tensile properties of road performance.

In this paper, special A-type sealant (special material type), special B-type sealant (special material type), 70# hot asphalt (heating construction type), SBS (Styrene–Butadiene–Styrene block copolymer)-modified asphalt (heating construction type), and room-temperature water-based sealant (normal-temperature construction type) commonly used in domestic laboratory tests were studied. Among them, type-A and type-B grouting pastes are two different types of grouting pastes with distinct components.

## 3. Test Results

### 3.1. Material Property Test

#### 3.1.1. High-Temperature Stability

(1) Softening-point test

**Figure 1:**
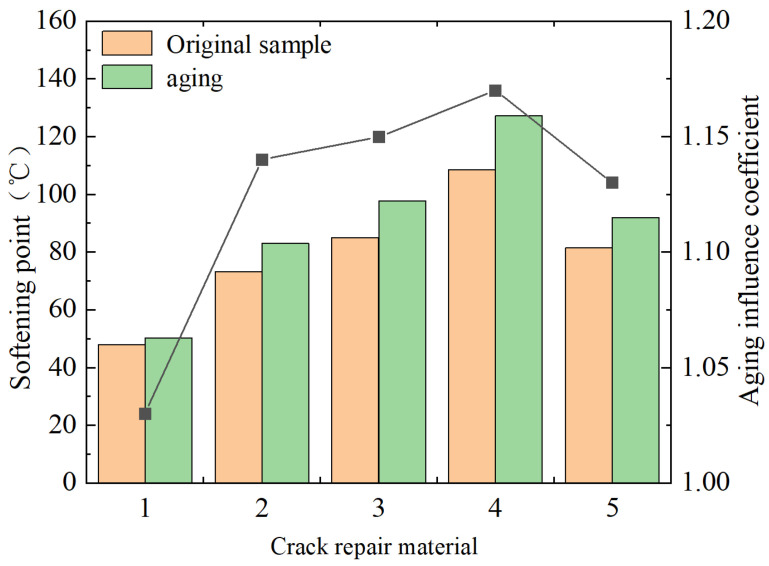
Softening-point test results. Note: (1) 70#, (2) SBS, (3) special A-type grouting sealant, (4) special B-type grouting sealant, and (5) water-based grouting sealant at normal temperature.

The operation shall be carried out by the requirements of Test Regulations for Asphalt and Asphalt Mixture of Highway Engineering (2011) [25]. Each material is molded into three parallel specimens for testing, and the same specimen is tested twice. If the coefficient of variation of the measured value is less than 8%, the average value is taken as the softening-point test result. The test results are shown in Figure 1.

As can be seen from the figure, there is a significant difference in the softening points of the five materials involved in this test, and the actual measurement data of the highest value exceeds twice that of the lowest value. The main influencing factors are the type and dosage of the polymer modifier [26]. The polymer forms a three-dimensional cross-linked network, significantly enhancing the material’s high-temperature resistance and cohesion. Polymer modifiers play a role of restraint and regulation in solid-state crystallization, enabling the crystal structure to resist thermal motion and allowing the crystal and the polymer network to jointly bear stress. The softening point of 70# hot asphalt obtained in this test is 47.9 °C, and when the temperature is raised to 60 °C, its form is completely transformed into liquid. The order of its high-temperature resistance from high to low is as follows: special type-B filling glue > special type-A filling glue > room-temperature water-based filling glue > SBS-modified asphalt > 70# hot asphalt.

The aging influence coefficient, *A*, can be used to reflect the specific anti-aging performance [27]:

(1) $$A=\frac{\mathrm{Test}\;\mathrm{value}\;\mathrm{of}\;\mathrm{aging}\;\mathrm{specimen}\;\mathrm{test}\;\mathrm{value}}{\mathrm{Test}\;\mathrm{value}\;\mathrm{of}\;\mathrm{the}\;\mathrm{original}\;\mathrm{specimen}}$$

The results of the aging simulation test show that the measured values of the softening point of the material at this time are greater than that of the initial material, and the aging influence coefficient of 1.15 is almost the same. The analysis shows that, under the action of a high-temperature thermal oxidation environment, the chemical reaction of oxygen absorption of the material components leads to this result.

(2) Fluidity test

**Figure 2:**
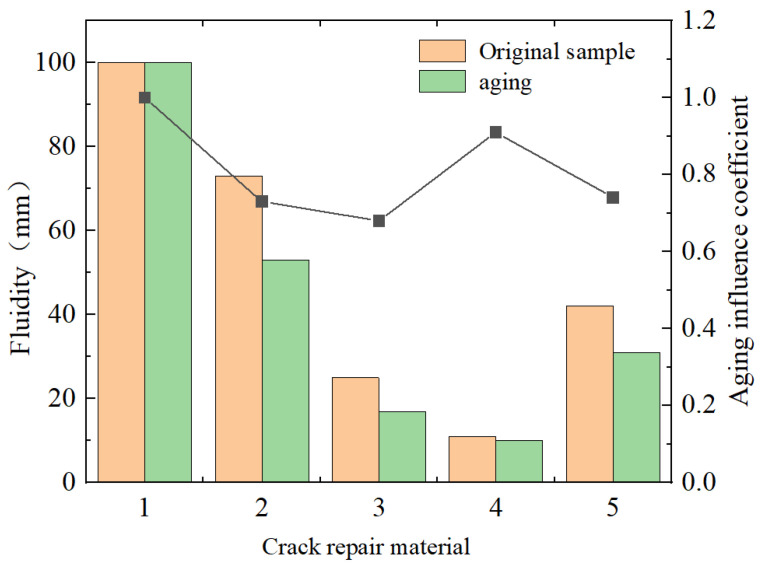
Fluidity test results. Note: (1) 70#, (2) SBS, (3) special A-type grouting sealant, (4) special B-type grouting sealant, and (5) water-based grouting sealant at normal temperature.

The discussion of the high-temperature flow of caulking materials focuses on various heating repair materials. For the test method, refer to “road heating type sealant (JT/T 740-2015)”. The test results are shown in Figure 2.

The fluidity size can reproduce the working state of the material under a high-temperature external environment in a more specific way. In general, the smaller the fluidity, the more prominent the corresponding high-temperature resistance of the material [28]. It can be seen from the chart that the lowest fluidity is the special type-B filling glue, and the order of high-temperature resistance is as follows: special type-B filling glue > special type-A filling glue > normal-temperature water-based filling glue > SBS-modified asphalt > 70# hot asphalt. The results of the aging simulation test show that the flow of various materials will have different behaviors under a high-temperature thermal oxidation environment, among which, the flow fluctuation is the largest, and the smallest is the special A-type filling glue and the special B-type filling glue, and the flow test data of the two decreased by about 30% and 10% respectively.

#### 3.1.2. Low-Temperature Crack Resistance

(1) Ductility test (5 °C)

**Figure 3:**
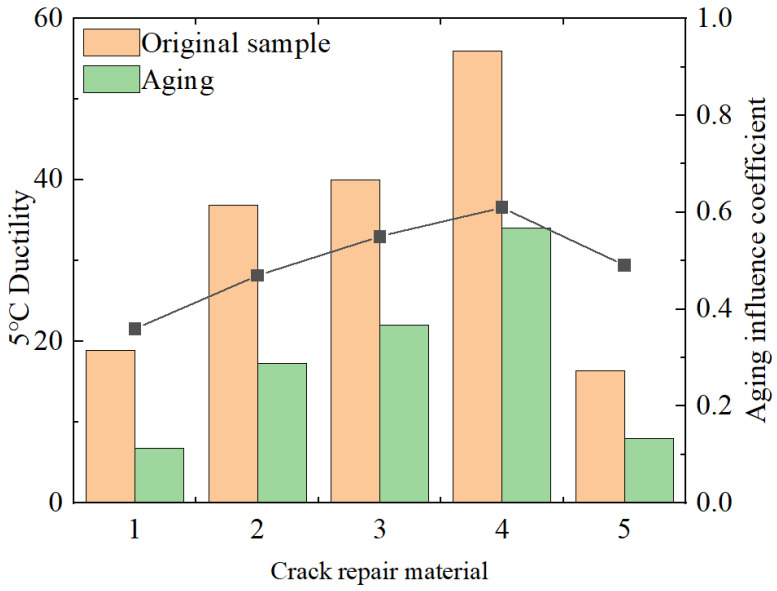
Ductility (5 °C) test results. Note: (1) 70#, (2) SBS, (3) special A-type grouting sealant, (4) special B-type grouting sealant, and (5) water-based grouting sealant at normal temperature.

The repair-material ductility test was carried out at 5 °C, and three parallel specimens of each material were formed for the test. If the coefficient of variation of the measured values of the three specimens was within 10%, the arithmetic mean value was taken as the measured value of the sample, and the test results are shown in Figure 3.

The variation in the low-temperature ductility test index values of materials has a high correlation with their low-temperature cracking performance. The ductility exhibited by the repair materials at a test temperature of 5 °C reflects their low-temperature crack resistance. The greater the ductility, the more prominent the low-temperature crack resistance of the corresponding material. As shown in the chart, the order of low-temperature crack resistance from high to low is as follows: special B-type sealant > special A-type sealant > SBS-modified asphalt > room-temperature water-based sealant > 70# hot asphalt. The low-temperature ductility of the first three materials is not significantly different and is about twice that of the latter two, indicating that they have better low-temperature resistance. The results of the aging simulation test show that at the same 5 °C temperature, the measured ductility values of the materials are all half of the initial values. Among them, the measured data of 70# hot asphalt are the worst, with a decrease of two-thirds, which also indicates that material aging has a significant impact on its low-temperature resistance.

(2) Elastic recovery test (5 °C)

**Figure 4:**
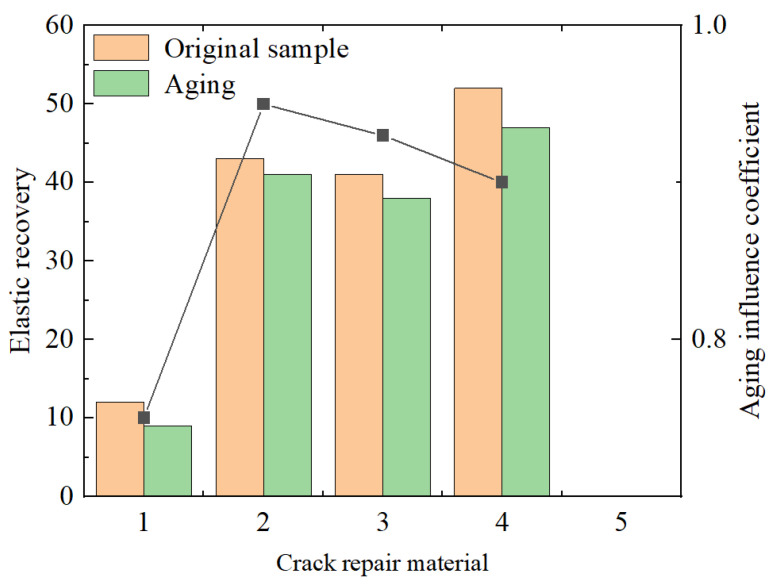
Elastic test results. Note: (1) 70#, (2) SBS, (3) special A-type grouting sealant, (4) special B-type grouting sealant, and (5) water-based grouting sealant at normal temperature.

The percentage of recoverable deformation after the penetration of the joint sealant material by a penetration ball is measured. The test method refers to the elastic recovery test of the joint sealant material standard. The test results are shown in Figure 4.

The elastic recovery rate refers to the ability of road asphalt joint sealant to restore its original shape under specific conditions. The higher the data value of the elastic recovery rate, the more prominent the low-temperature resistance performance of the corresponding material. As can be seen from the figure, the low-temperature crack resistance of the five repair materials from high to low is as follows: special B-type joint sealant > SBS-modified asphalt > special A-type joint sealant > 70# hot asphalt > room-temperature water-based joint sealant. The results of the aging simulation test show that the measured values of the elastic recovery rate of the materials at this time are all less than the initial materials, and the elastic performance of all materials slightly decreases in the thermal aging environment.

#### 3.1.3. Resistance to Foreign Body Insertion

**Figure 5:**
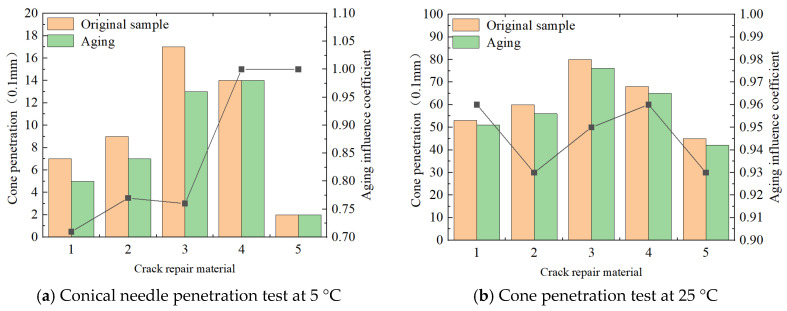
Cone penetration test results. Note: (1) 70#, (2) SBS, (3) special A-type grouting sealant, (4) special B-type grouting sealant, and (5) water-based grouting sealant at normal temperature.

The resistance of the material to the intrusion of these foreign debris is usually measured by the cone-type needle penetration test. The results are shown in Figure 5.

In the cone penetration test, the smaller the experimental data of the general cone penetration, the more prominent the anti-foreign body invasion performance of the corresponding material. As can be seen from the figure, the anti-foreign body intrusion test data are ranked from high to low: room-temperature water-based sealant > 70# hot asphalt > SBS-modified asphalt > special B-type sealant > special A-type sealant, but the values of various materials meet the technical requirements of ordinary sealant, which also indicates that this performance difference is not prominent. In addition, the penetration of conical needles obtained at a low temperature of 5 °C is much lower than that at a normal temperature of 25 °C, which reflects that at a low temperature, the hardness of the material will be better, resulting in more difficulty for foreign bodies to squeeze. Aging simulation test results show that, under the two temperature conditions, the cone penetration-test values obtained by all materials in the test are reduced. It is analyzed that, after aging, the molecular structure of materials will also change correspondingly, as reflected in the enhanced hardness performance, resulting in more difficult foreign body intrusion. However, it should be noted that the influence coefficient of aging is not prominent. It is only slightly smaller than 1.0, which also means that, under high-temperature thermal oxidation conditions, the influence on cone penetration is small.

#### 3.1.4. Corrosion Resistance

**Figure 6:**
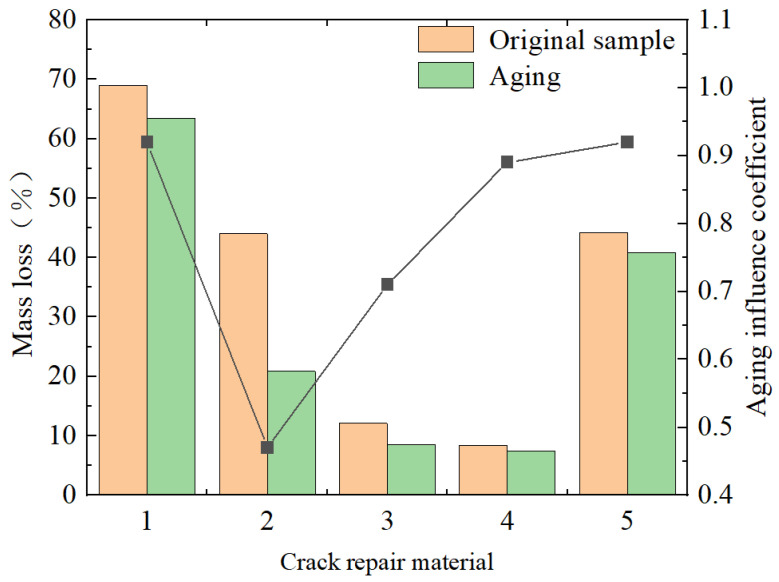
Corrosion resistance test results. Note: (1) 70#, (2) SBS, (3) special A-type grouting sealant, (4) special B-type grouting sealant, and (5) water-based grouting sealant at normal temperature.

Corrosion resistance test Three parallel specimens of each material are formed respectively for testing. If the coefficient of variation of the measured values of the three specimens is within 8%, the arithmetic mean value is taken as the measured value of the sample. The test results are shown in Figure 6.

(2) $$\alpha =\frac{m_{1}-m_{2}}{m_{1}}\times 100\%$$

where

*α*—mass loss (%);
*m*_1_—initial sample mass (g);
*m*_2_—sample residual mass (g).

The smaller the mass loss test value, the higher the corrosion resistance of the corresponding material. It can be seen from Figure 6 that the quality-loss values of the five materials measured in this test differ greatly, among which the quality loss is relatively small for the special B-type sealant and the special A-type sealant. The mass loss of 70# hot asphalt is more than 60%, resulting in poor corrosion resistance. The corrosion resistance of the materials can be compared in the order from high to low as follows: special type-B filling glue > special type-A filling glue > SBS-modified asphalt > room-temperature water-based filling glue > 70# hot asphalt. The results of the aging simulation show that high-temperature thermal oxidation environments have a significant influence on the corrosion resistance of materials, and the influence level of different materials is different. Among them, after aging, the corrosion resistance of special B-type sealant and special A-type sealant decreased by 10% and 30%, and the thermal oxygen aging resistance was good, meeting the structural performance requirements of crack repair, while the SBS-modified asphalt decreased by 53%, and the 70# hot asphalt was very little affected, as the decrease was only 7%. The analysis shows that the material components have a greater quality loss when facing some corrosive substances than plateau samples, and the corrosion is not obvious after aging. However, the normal-temperature water-based sealant was partially aging due to the previous water evaporation treatment, so it was then subjected to hot oxygen aging treatment, but the quality loss was not obvious, and the reduction was only 8%.

### 3.2. Road Performance Test

#### 3.2.1. Shear Resistance

**Figure 7:**
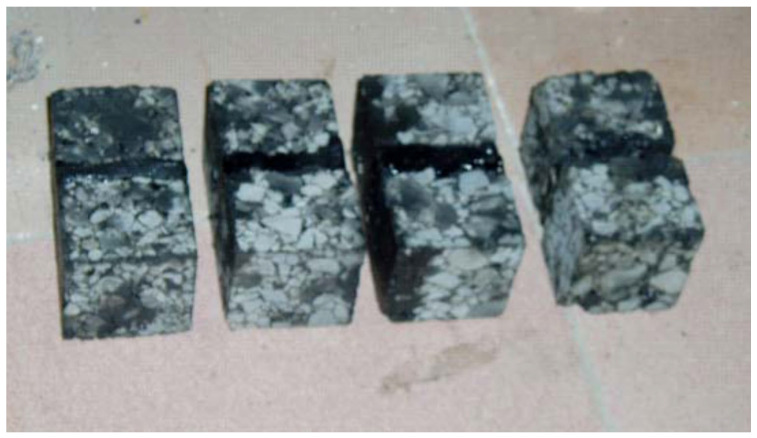
Direct shear test specimen.

The standard rutting plate is cut to obtain several small test blocks that meet the test conditions, and the specification is designed to be 50 × 50 × 32 mm. At the same time, the test block needs to be processed, and the two sides of the square are left hair and cut light state, respectively. Set the test blocks in two groups with vernier calipers, form 5 mm empty slots relative to the hairy side, seal the gaps on both sides and the bottom, and fill in the repair material after it is thoroughly formed and stable. Then, wait for a while until it solidifies, as shown in Figure 7, and clean up the spilled material or other debris on the surface of the test block. At the same time, the length, *L*_1_, and width, *L*_2_, of the material seam are measured using a caliper.

For each material, 30 specimens were made through the above process (it should be noted here that the original and aging specimens are counted as one material, and the drawing test is also treated in the same way), and they were divided into two groups, each of which was allocated 15 specimens. Note that the water infiltration into the interface crevices will reduce the friction coefficient and mechanical bite force, thereby weakening the shear-resistance performance. The first group was not treated, and the second group was first placed in water, immersed for 4 d, wiped clean after removal, and then left to dry for 24 h. Each group was divided into three groups. There were five specimens in each group, and then the groups were placed in environmental chambers with different temperatures for a total of 4 h. The temperature conditions were set at 0 °C, 20 °C, and 50 °C, respectively. These specimens were subjected to vertical compressive stress at a rate of 10 mm per minute. After the specimen was destroyed, the maximum vertical shear stress value, *P_J_*, was recorded during the process.

The direct shear strength is calculated according to Formula (3), *Q*_J_:

(3) $$Q_{J}=\frac{P_{J}}{L_{1}\times L_{2}}\times 10^{3}$$

where

*Q_J_*—direct shear strength (MPa);
*P_J_*—maximum vertical shear stress (kN);
*L*_1_—specimen length (mm);
*L*_2_—specimen width (mm).

**Figure 8:**
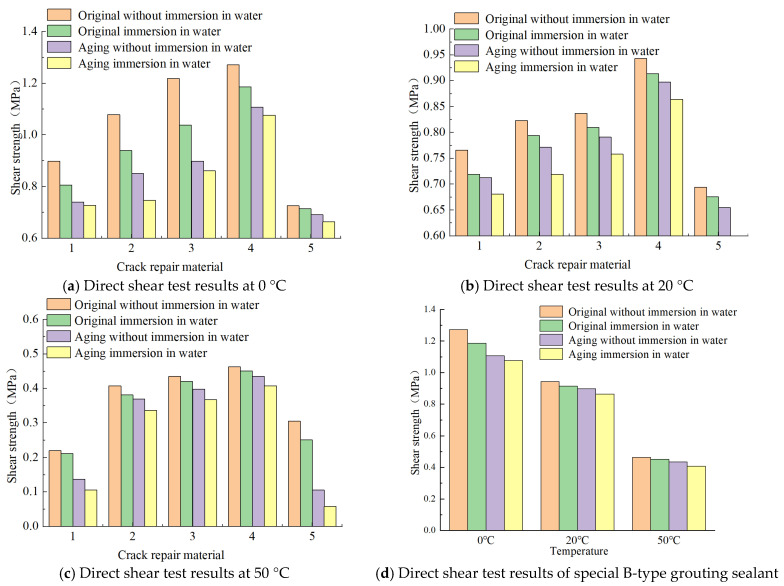
Direct shear test results. Note: (1) 70#, (2) SBS, (3) special A-type grouting sealant, (4) special B-type grouting sealant, and (5) water-based grouting sealant at normal temperature.

The average values of five specimens were taken as test results, as shown in Figure 8.

It is not difficult to find that the direct shear strength of the material decreases significantly after the temperature increases, as is consistent with the properties of asphalt materials. Moreover, if the given external temperature conditions are 0 °C and 20 °C, the sequence of direct shear strength of each material measured at this time does not change significantly, and is about the same, from high to low. The order is special B-type filling glue > special A-type filling glue > SBS-modified asphalt > 70# hot asphalt > normal-temperature water-based filling glue.

For 70# hot asphalt, it has complete fluidity at the temperature of 50 °C, so its direct shear strength measured value is lower than that of other materials after aging, meaning that this kind of material is completely unsuitable for repair in high-temperature environments. At the same time, although the original sample of water-based filling glue at normal temperature has good direct shear strength, this index will drop sharply once water penetration or aging occurs. Special type-B sealant, special type-A sealant, and SBS-modified asphalt still maintain high shear strength under high-temperature conditions and are suitable for use in high-temperature areas in summer.

#### 3.2.2. Tensile Property

The tensile properties of crack-repair structures under external loads at different temperatures were measured via the drawing test [29]. The external load here can be caused either by vehicle traffic or by the contraction of the asphalt mixture.

The procedure of this test is consistent with the previous direct shear test, only the vertical tensile stress should be provided when the stress is loaded, and the vertical maximum tensile stress value in the process should be recorded in detail, *P_L_*.

The drawing strength is calculated according to Formula (4), *Q_L_*:

(4) $$Q_{L}=\frac{P_{L}}{L_{1}\times L_{2}}\times 10^{3}$$

*Q_L_*—tensile strength (MPa);
*P_L_*—maximum vertical tensile stress (kN);
*L*_1_—specimen length (mm);
*L*_2_—specimen width (mm).

**Figure 9:**
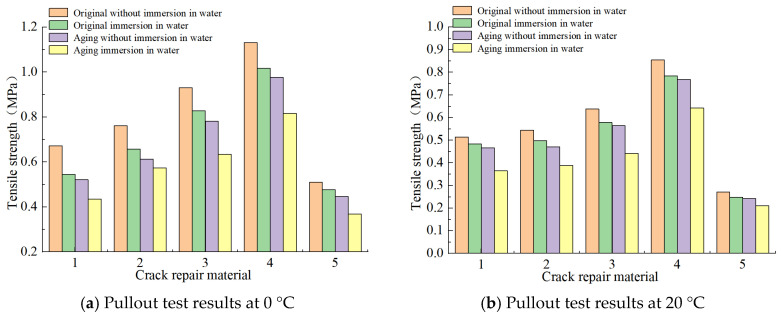

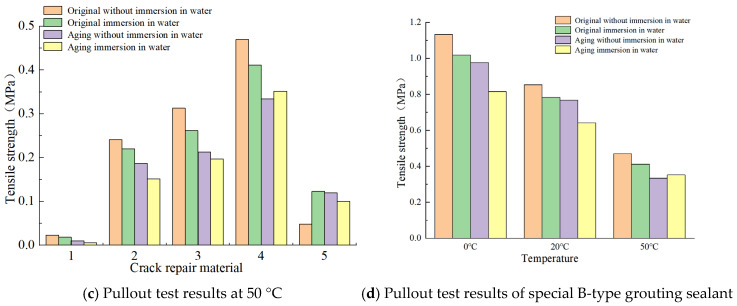
Pullout test results. Note: (1) 70#, (2) SBS, (3) special A-type grouting sealant, (4) special B-type grouting sealant, and (5) water-based grouting sealant at normal temperature.

The average values of five specimens were taken as test results, as shown in Figure 9:

The data show that the drawing strength of the material decreases significantly after the temperature increases. If the given external temperature conditions are 0 °C and 20 °C, there is no significant change in the order of drawing strength of each material. From high to low, the order is as follows: special B-type filling glue > special A-type filling glue > SBS-modified asphalt > 70# hot asphalt > normal-temperature water-based filling glue.

At a high temperature of 50 °C, the drawing strength of various repair materials is similar to that of direct shear strength, and the value of special B-type filling glue and special A-type filling glue is higher, and either is recommended to be chosen. The drawing strength of SBS-modified asphalt can also meet the construction requirements and can be used as a secondary choice. In various tests, the water-based filling glue at normal temperature shows that its high-temperature drawing strength is high, but it cannot meet the required direct shear strength, so it cannot meet the requirements of asphalt pavement repair. Similarly, 70# hot asphalt can be excluded according to the test results. It is worth noting that, under the conditions of aging or immersion, it will inevitably affect the direct shear strength and drawing strength, and in both cases, the influence degree of aging is greater if the aging and then the immersion significantly accelerate the strength-decay level. After repairing cracks, aging will inevitably occur with the passage of time and other factors. At this time, attention should be paid to the waterproof function of the joint structure to delay the further aging of the joint structure.

## 4. Analysis of Stress State of Crack-Repair Structure

### 4.1. Finite-Element Model of Asphalt-Pavement Crack-Repair Structure

#### 4.1.1. Structural Layer Parameter

In this paper, by using ABAQUS 2021 finite-element analysis software to discuss the adopted pavement structural model, the four-layer-type semi-rigid base-layer asphalt pavement structural form with high generality is selected [30]. The material parameters of each structural layer of the pavement are taken according to the values commonly used in the “Highway Asphalt Pavement Design Code” “Appendix D Material Design Parameters” and related information, as shown in Table 1.

#### 4.1.2. Illustration of Load Effects

**Figure 10:**
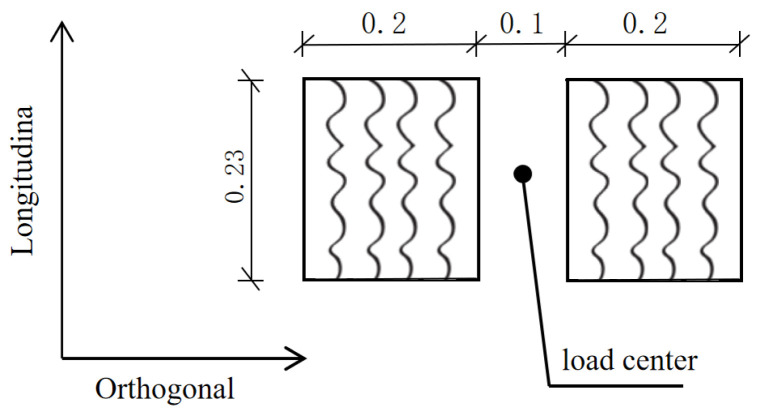
Equivalent double-wheel load plan (unit: cm).

**Figure 11:**
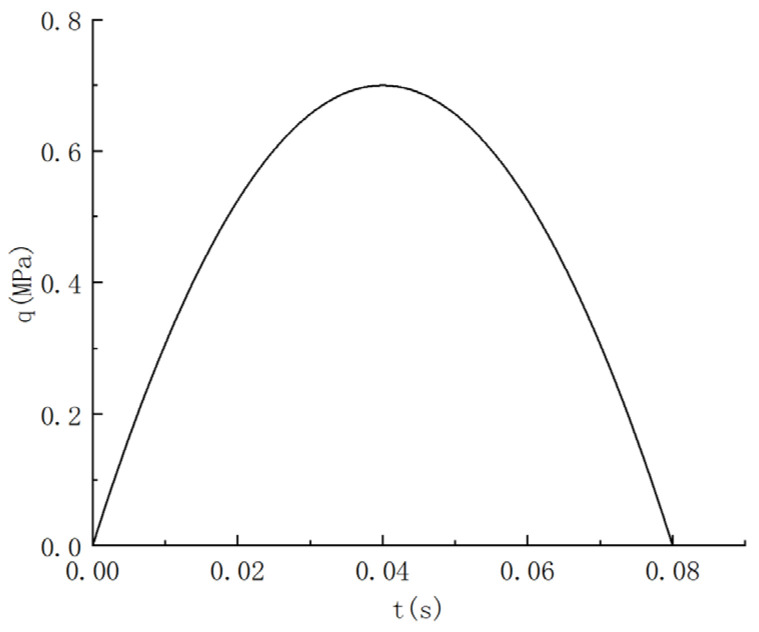
Load strength over time.

**Figure 12:**
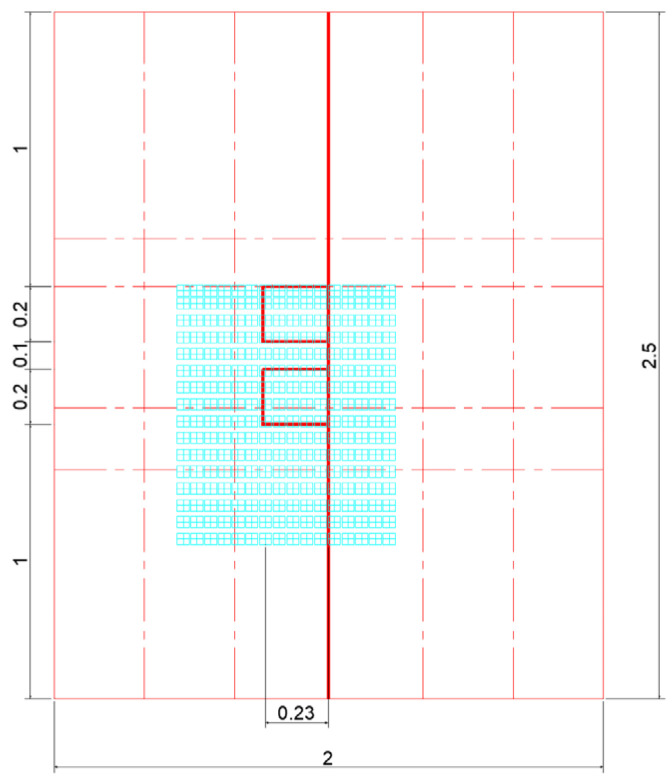
Top surface dimensions and load action area of the pavement structure.

Set “static load” and “dynamic load” as two working conditions, corresponding to the vehicle load “static” and “traveling”, respectively. The two conditions of “static load” and “dynamic load” correspond to the effects of vehicle loads on the internal forces of the repair structure under the two states of “stationary” and “traveling”, respectively. Static load: The wheel load is equivalent to two rectangular uniform loads [31]; see Figure 10. Dynamic loads: Half-sine loads are set; see Figure 11. The dimensions of the top surface of the pavement structure and the loaded area are shown in Figure 12.

**Figure 13:**
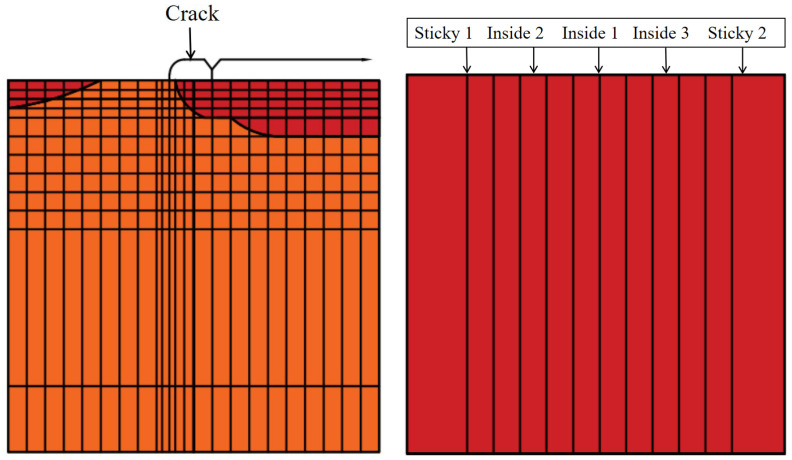
Crack and analysis interface location.

From the preliminary analysis results, it can be seen that when the load acts on the boundary of one side of the crack, the specific condition can be referred to in the above figure; at this time, the internal force is the largest, so this position will be used as the selection point of the load action for the subsequent calculation and analysis. Figure 13 gives the location of the crack in the model and the location of the analyzed interface, respectively.

### 4.2. Finite-Element Calculation Results

The stress maxima all appear at the same location, i.e., the bonding surface between the repair material and the wall, that is, Sticky 1 or Sticky 2 in the above figure; in other words, the stress damage interface has a higher probability of appearing at these two points as well. Therefore, in this paper, only the force state on the bonding surface (Sticky 1 or Sticky 2) is considered in the analysis. Table 2 details the calculated values of maximum stress values for the repair structure under different factor conditions.

Table 2 shows the maximum stress value of the crack-repair structure under the most unfavorable conditions at different temperatures, and such a value can be regarded as the theoretical maximum stress value of the repair structure, which requires that the measured stress value of the repair material be greater than the theoretical maximum stress value to ensure the strength of the grouting structure, and the shear and compressive strengths of the indoor test of the repair material can be obtained by the previous part of the road performance test. Figure 11 shows the straight shear test results of the repair material, which are compared with the maximum shear stress in Table 2 and Table 3.

Similarly, Figure 12 shows the test results of the drawing performance of the repair material, and these results are compared with the maximum tensile stress in Table 2 and Table 4.

### 4.3. Analysis of Calculation Results

In the low-temperature and room-temperature state, repair materials in the grouting structure can withstand the role of shear stress; however, in the high-temperature 50 °C conditions, 70# asphalt and room-temperature water-based grouting adhesive in the aging effect of its shear strength is less than the theoretical maximum shear stress, so it cannot withstand pavement structure and may be subjected to the role of shear stress. Thus, these two materials, if used in high-temperature areas in the summer in the role of vehicular loading, are likely to experience shear damage.

The tensile strength of five kinds of repair materials under room-temperature conditions is far from meeting the requirements of tensile properties. However, in 0 °C conditions, 70# asphalt and room-temperature water-based grouting adhesive tensile strength–stress ratio cannot meet the requirements, so low-temperature tensile performance is poor. In 50 °C high-temperature conditions, 70# asphalt in high-temperature conditions is very easily subjected to tensile crack damage, and its use should be avoided for summer high-temperature areas.

For dedicated B-type grouting adhesive and dedicated A-type grouting adhesive, the shear strength and maximum shear stress ratio in high-temperature and low-temperature conditions are more than 1.4, and the shear performance is excellent. SBS-modified asphalt is ranked second; in high-temperature, low-temperature, and room-temperature conditions, its shear strength and maximum shear stress ratio are more than 1, and its tensile performance advantage is obvious, as verified by the order of the shear and the tensile properties obtained from the indoor test.

## 5. Conclusions

In this paper, five representative repair materials from China are selected to carry out indoor performance test research and are combined with finite-element mechanical analysis results for comparative verification. The main conclusions are as follows:

(1) Special B-type crack sealant (special B-type crack sealant) shows excellent shear performance, tensile performance, and durability under extreme working conditions. Under the high-temperature condition of 50 °C, its measured ratio of shear strength to theoretical maximum shear stress reaches 2.25, which is significantly higher than that of other materials, indicating that it is suitable for heavy traffic roads (such as highways and urban expressways) in high-temperature areas in summer. In the 0 °C low-temperature environment, its tensile strength stress ratio far exceeds that of 70# hot asphalt and room-temperature water-based grouting adhesive, which can effectively inhibit the expansion of low-temperature shrinkage cracks. After thermo-oxidative aging, the shear strength only decreased by about 10%, and the corrosion resistance (mass loss rate < 5%) and the resistance to foreign body embedding ability were better than other materials, verifying the feasibility of its long-life repair.
(2) SBS (Styrene-Butadiene-Styrene block copolymer)-modified asphalt in heating construction materials, at 20 °C room temperature, has a shear strength and tensile strength that can meet the needs of the second level of highway, but its high-temperature performance is significantly weaker than the special materials, so it is recommended to be used for medium traffic volume of the road cyclical repair. Despite the low cost of 70# hot asphalt, because of its high-temperature shear strength (about 1/3 of the special materials), it is recommended for use in medium traffic volume road cyclical repair. However, its high-temperature shear strength and low-temperature tensile strength (0 °C after aging at 0.95 MPa) are lower than the theoretical stress threshold, making it only suitable for low-traffic feeder roads or temporary repair.
(3) Room-temperature construction materials, such as water-based grouting adhesives, demonstrate good wettability under wet conditions. Their high-temperature shear and tensile strength (3.08 MPa) are insufficient to withstand dynamic vehicle loads. They exhibit poor low-temperature elastic recovery rates (<40%) and aging resistance, with strength decay exceeding 50%. Due to these limitations, these materials are only suitable for emergency seepage control or short-term crack closure after rain or snow events.

## Figures and Tables

**Table 1:** Parameter of various structural layers of road surface.

Structural Layer	Material	Thickness (m)	Temperature (°C)	Elastic Modulus (Mpa)
Surface layer	Asphalt concrete	0.18	0	2200
			20	1200
			50	500
Base	Cement-stabilized macadam	0.30	20	4500
Subbase	Lime-fly ash stabilized soil	0.30		800
Soil foundation	Backfill soil	5.00		50
Inside the crack	Repair materials	0.18	0	280
			20	150
			50	70

**Table 2:** Maximum stress values that crack-repair structures may experience.

State of Temperature	Maximum Shear Stress (Mpa)	Maximum Tensile Stress (Mpa)	Maximum Compressive Stress (Mpa)
Low-temperature state	0.603	0.4580	1.249
Normal-temperature state	0.576	0.0489	1.137
High-temperature conditions	0.206	0.0393	0.237

**Table 3:** Measured shear strength to maximum shear stress ratio.

Material	Temperature (°C)	Measured Shear Strength (Mpa)/Theoretical Maximum Shear Stress (Mpa)			
		Original Without Immersion in Water	Original Immersion in Water	Aging Without Immersion in Water	Aging Immersion in Water
70#	0	1.49	1.33	1.23	1.21
	20	1.33	1.25	1.24	1.18
	50	1.07	1.03	0.67	0.51
SBS	0	1.80	1.55	1.41	1.23
	20	1.43	1.38	1.32	1.25
	50	1.97	1.85	1.79	1.64
Special A-type grouting sealant	0	2.02	1.72	1.49	1.43
	20	1.45	1.41	1.37	1.32
	50	2.11	2.04	1.93	1.79
Special B-type grouting sealant	0	2.11	1.97	1.84	1.78
	20	1.64	1.59	1.56	1.50
	50	2.25	2.19	2.11	1.98
Water-based grouting sealant at normal temperature	0	1.20	1.18	1.15	1.01
	20	1.20	1.17	1.14	1.03
	50	1.48	1.22	0.51	0.28

**Table 4:** Measured ratio of tensile strength to maximum tensile stress.

Material	Temperature (°C)	Measured Tensile Strength (Mpa)/Theoretical Maximum Tensile Stress (Mpa)			
		Original Without Immersion in Water	Original Immersion in Water	Aging Without Immersion in Water	Aging Immersion in Water
70#	0	1.47	1.19	1.14	0.95
	20	10.49	9.86	9.51	7.45
	50	0.59	0.46	0.26	0.15
SBS	0	1.66	1.44	1.34	1.25
	20	11.08	10.16	9.61	7.91
	50	6.18	5.64	4.79	3.87
Special A-type grouting sealant	0	2.03	1.81	1.71	1.38
	20	13.02	11.82	11.53	9.00
	50	8.03	6.72	5.46	5.05
Special B-type grouting sealant	0	2.47	2.22	2.13	1.78
	20	17.43	16.00	15.67	13.10
	50	12.05	10.56	8.56	7.74
Water-based grouting sealant at normal temperature	0	1.11	1.04	0.98	0.80
	20	5.55	5.06	4.94	4.31
	50	3.79	3.75	3.08	2.56

## Data Availability

The original contributions presented in this study are included in the article. Further inquiries can be directed to the corresponding author.
